# Simple Synthesis of Molybdenum Disulfide/Reduced Graphene Oxide Composite Hollow Microspheres as Supercapacitor Electrode Material

**DOI:** 10.3390/ma9090783

**Published:** 2016-09-20

**Authors:** Wei Xiao, Wenjie Zhou, Tong Feng, Yanhua Zhang, Hongdong Liu, Liangliang Tian

**Affiliations:** Research Institute for New Materials Technology, Chongqing University of Arts and Sciences, Yongchuan, Chongqing 402160, China; wenjie_zhou@aliyun.com (W.Z.); fengtong022754@126.com (T.F.); lhd0415@126.com (H.L.); tianll07@163.com (L.T.)

**Keywords:** molybdenum disulfide, reduced graphene oxide, hollow microsphere, supercapacitor, energy storage, hydrothermal synthesis

## Abstract

MoS_2_/RGO composite hollow microspheres were hydrothermally synthesized by using SiO_2_/GO microspheres as a template, which were obtained via the sonication-assisted interfacial self-assembly of tiny GO sheets on positively charged SiO_2_ microspheres. The structure, morphology, phase, and chemical composition of MoS_2_/RGO hollow microspheres were systematically investigated by a series of techniques such as FE-SEM, TEM, XRD, TGA, BET, and Raman characterizations, meanwhile, their electrochemical properties were carefully evaluated by CV, GCD, and EIS measurements. It was found that MoS_2_/RGO hollow microspheres possessed unique porous hollow architecture with high-level hierarchy and large specific surface area up to 63.7 m^2^·g^−1^. When used as supercapacitor electrode material, MoS_2_/RGO hollow microspheres delivered a maximum specific capacitance of 218.1 F·g^−1^ at the current density of 1 A·g^−1^, which was much higher than that of contrastive bare MoS_2_ microspheres developed in the present work and most of other reported MoS_2_-based materials. The enhancement of supercapacitive behaviors of MoS_2_/RGO hollow microspheres was likely due to the improved conductivity together with their distinct structure and morphology, which not only promoted the charge transport but also facilitated the electrolyte diffusion. Moreover, MoS_2_/RGO hollow microsphere electrode displayed satisfactory long-term stability with 91.8% retention of the initial capacitance after 1000 charge/discharge cycles at the current density of 3 A·g^−1^, showing excellent application potential.

## 1. Introduction

To meet the rapidly increasing energy demand, supercapacitors have emerged as a new kind of advanced energy storage device owing to their high power capability, quick charge/discharge rate, long cycle life, and simple configuration [1,2,3,4]. According to the charge storage mechanism, supercapacitors are generally divided into two groups [1,2,3,4]. One is electric double-layer capacitors (EDLCs), which works depending on the charge separation at the electrode/electrolyte interface and the active matters incorporated in EDLC electrodes are mainly made of nanostructured carbonaceous materials with large surface area like activated carbon, carbon nanotubes, graphene, and carbide-derived carbon [1,2,3,4]. The other is pseudocapacitors, and the energy storage within which relies on the fast and reversible faradaic reactions occurring in the active electrode materials such as metal oxides, metal sulfides, metal hydroxides, conducting polymers, and their hybrid composites [1,2,3,4]. During the past decade, as a promising type of supercapacitor electrode materials, layered transition-metal dichalcogenides (TMDs), including MoS_2_, VS_2_, SnS_2_, WS_2_, etc., have received more and more attention due to their exciting physicochemical properties [5,6,7,8,9,10]. Among them, MoS_2_ is a representative family member of TMDs, since its crystal consists of the metal Mo layers sandwiched between double sulfur layers, which is then stacked together via van der Waals forces to form a layered structure like graphite [5,6]. Such a special structure and its excellent mechanical and electric properties make it become one of the hottest materials in the research area of supercapacitors [5,6]. For instance, Wang et al. reported a 3D flower-like MoS_2_ nanostructure with the specific capacitance of 168 F·g^−1^ at the discharge current density of 1 A·g^−1^ [11]. Zhou et al. synthesized flower-like MoS_2_ nanospheres through a hydrothermal process, which delivered a specific capacitance of 122 F·g^−1^ at 1 A·g^−1^ [12]. Ramadosset et al. prepared a nanostructured mesoporous MoS_2_ electrode material and it released a specific capacitance of 124 F·g^−1^ at 1 A·g^−1^ [13]. More recently, Wang et al. developed hollow MoS_2_ nanospheres by means of a template method and the specific capacitance of the corresponding electrode was about 130 F·g^−1^ at 1 A·g^−1^ [3]. Ilanchezhiyan et al. fabricated a spherically clustered MoS_2_ nanostructure and the specific capacitance of such electrode reached to around 160 F·g^−1^ at 1 A·g^−1^ [14]. Besides bare MoS_2_ materials, MoS_2_-based composites have been explored and utilized for supercapacitor applications as well. For example, MoS_2_/C hybrid material was one-pot hydrothermally synthesized and its specific capacitance was about 180 F·g^−1^ at 1 A·g^−1^ when used as a supercapacitor electrode material [6]. Heterostructured MoS_2_/SnS_2_ and MoS_2_/SnO_2_ composites were also developed through hydrothermal and microwave-assisted hydrothermal reactions and their specific capacitance was found to be about 120 and 159 F·g^−1^ and at 1 A·g^−1^, respectively [15,16]. In most cases, the specific capacitance of MoS_2_-based supercapacitor electrode materials does not exceed 200 F·g^−1^, which is much lower than the theoretical capacitance of MoS_2_. It is assumed that that the intrinsic poor electric conductivity of MoS_2_ and the serious stacking and agglomeration of two-dimensional MoS_2_ sheets during the synthetic process are the key reasons [17]. Because the former limits the transport of electrons and ions in the electrode matrix, while the later severely reduces the specific surface area of the active electrode materials, thus resulting in relatively inferior electrochemical behaviors. Consequently, increasing the electric conductivity of MoS_2_-based composite materials together with constructing hierarchical architecture with high specific surface area through a simpleand cost-effective strategy to boost their supercapacitive performances seems to be a feasible way but remains a challenging task. 

**Figure 1:**
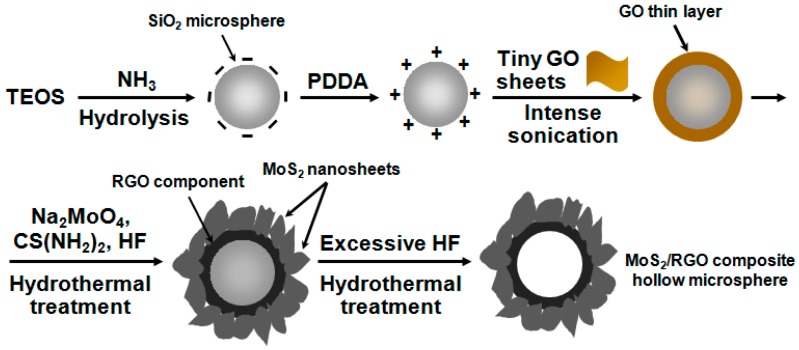
Schematic illustration of the preparation of MoS_2_/RGO composite hollow microspheres.

Graphene, a monolayer of graphite, has emerged as one of the most popular star materials because of its fascinating properties and functions [18,19,20,21]. Specifically, its extraordinary electric conductivity and long-term chemical stability make it widely applicable in the fields of energy storage and conversion, and hence lots of graphene or reduced graphene oxide (RGO)-containing composites with reinforced electrochemical behaviors have been explored [5,17,22]. Typically, MoS_2_/graphene and MoS_2_/RGO hybrid materials were fabricated by different methods and the corresponding electrodes gave pronounced supercapacitive performances [23,24,25]. For instance, layered MoS_2_ was directly deposited on RGO sheets by microwave-assisted synthesis approach, MoS_2_ nanosheet−graphenenanosheet hybrid films were developed via layer-by-layer techniques, and in situ decoration of laser-induced graphene with MoS_2_ was accomplished by spin-coating MoS_2_ nanoflakes onto polyimide foil followed by graphenization of the polymer using a CO_2_ laser-writing process. In recent years, interfacial self-assembly of graphene oxide (GO) sheets on solid substrates has been proven to be an effective methodology to synthesize GO and RGO-containing hybrid materials [18,19]. We have also pioneered the sonication-assisted interfacial self-assembly of tiny graphene oxide (GO) sheets on cationic polyelectrolyte-modified SiO_2_ colloids via electrostatic interaction, leading to the formation of GO thin layer encapsulated SiO_2_ microspheres [20,21]. The resultant SiO_2_/GO composite microspheres possessed splendid water dispersity, which enabled them to be readily modified and functionalized in the following steps. In the present work, we make full use of this point and employ them as a template to fabricate MoS_2_/RGO composite hollow microspheres with hierarchically porous architecture. As schematically presented in Figure 1, the SiO_2_/GO microspheres underwent a hydrothermal process in the presence of sodium molybdate, thiourea, and a small amount of hydrofluoric acid, and during such hydrothermal reaction, the deposition, growth, and crystallization of MoS_2_ on substrate microspheres and the reduction of GO to RGO component were simultaneously fulfilled in one pot, yielding SiO_2_/RGO/MoS_2_ microspheres. Afterwards, their SiO_2_ inner core was totally etched by excessive HF in another hydrothermal process, giving rise to the generation of the final product of MoS_2_/RGO composite hollow microspheres. When used as supercapacitor electrode material in a three-electrode system, they delivered a maximum specific capacitance of 218.1 F·g^−1^ at the discharge current density of 1 A·g^−1^ with satisfactory long cycling durability over 1000 cycles, exhibiting excellent application potential.

## 2. Experimental Section

### 2.1. Materials and Reagents

Tetraethyl orthosilicate (TEOS), ammonium hydroxide NH_3_·H_2_O (25 wt%), poly(diallyldimethylammonium) chloride (PDDA) with average molecular weight of 200,000~350,000, sodium molybdatedihydrate Na_2_MoO_4_·2H_2_O, thiourea CS(NH_2_)_2_, hydrofluoric acid HF (40 wt%), acetylene black, polyvinylidene fluoride (PVDF), *N*-methyl-2-pyrrolidone (NMP), and potassium hydroxide KOH were purchased from Sinopharm Chemical Reagent Co., Ltd. (Shanghai, China). Commercial GO sheets with the lateral size not more than 200 nm were bought from Nanjing JCNANO Technology Co., Ltd. (Nanjing, China). Nickel foam was provided by KunshanJiayisheng Electronics Co., Ltd. (Kunshan, China). All the chemicals were of analytical grade and used as received. Milli-Q water (resistivity, 18.2 MΩ·cm) was employed throughout this work.

### 2.2. Synthesis of SiO_2_/GO Composite Microspheres

SiO_2_ colloids were firstly synthesized according to a modified Stöber method reported previously [26,27]. In a typical procedure, 200 mL of ethanolic solution containing 0.08 mol TEOS was suddenly mixed with another 200 mL of ethanolic solution containing 0.16 mol ammonia and 6.8 mol water under moderate stirring, and the reaction was allowed to proceed overnight to produce SiO_2_ colloidal microspheres, which were then collected by centrifugation, washing, and drying. Afterwards, 1 g of as-prepared SiO_2_ microspheres were dispersed in 100 mL of water with the aid of sonication. The resulting suspension was slowly added into 100 mL of aqueous PDDA solution (1 wt%), and the mixture was vigorously stirred for more than 16 h to give positively charged SiO_2_ microspheres, which were harvested by centrifugation, washing, and drying. Subsequently, 120 mL of aqueous suspension of positively charged SiO_2_ microspheres (4 mg·mL^−1^) was dropwise added into another 120 mL of aqueous suspension of tiny GO sheets (0.4 mg·mL^−1^) within 30 min under violent sonication (160 W). The resulting mixture was successively sonicated for another 30 min to ensure the sufficient interfacial self-assembly of tiny GO sheets on the positively charged SiO_2_ microspheres through electrostatic interaction, thus yielding GO wrapped SiO_2_ microspheres (i.e., the SiO_2_/GO composite microspheres), which were then isolated from the unreacted GO sheets by centrifugation at a relatively low speed of 8000 rpm for 5 min. Finally, the obtained yellow-brown precipitates were dried in a vacuum oven after washing with copious water and centrifugation.

### 2.3. Synthesis of MoS_2_/RGO Composite Hollow Microspheres

80 mg of as-fabricated SiO_2_/GO microspheres were ultrasonically dispersed in 55 mL of water, followed by addition of 5 mL of aqueous solution containing 155 mg of Na_2_MoO_4_·2H_2_O, 243 mg of thiourea and a small amount of HF (30 μL, 40 wt%) under sonication to form a homogeneous reaction mixture. Then, it was transferred into a Teflon-lined stainless autoclave with the capacity of 100 mL and sealed to heat at 200 °C for 24 h. After this hydrothermal process, MoS_2_ was grown on the substrate microspheres and the GO component was simultaneously reduced to RGO, leading to the generation of SiO_2_/RGO/MoS_2_ microspheres. Subsequently, they were washed with abundant water and dispersed in 30 mL of water mixed with 250 μL of HF (40 wt%). The reaction mixture was then transferred into a Teflon-lined stainless autoclave with the capacity of 50 mL and subjected to further hydrothermal treatment at 180 °C for 12 h to remove the SiO_2_ inner core. As such, the final solid product of MoS_2_/RGO composite hollow microspheres were simply synthesized, which was harvested by washing, centrifugation, and drying. As a comparison, contrastive bare MoS_2_ microspheres were hydrothermally prepared in the absence of SiO_2_/GO microspheres, and the synthetic conditions were almost the same as those for fabrication of SiO_2_/RGO/MoS_2_ microspheres. Moreover, bare RGO material was obtained by hydrothermal treatment of aqueous suspension of tiny GO sheets (0.5 mg·mL^−1^) at 200 °C for 24 h.

### 2.4. Characterizations

Field emission scanning electron microscopy (FE-SEM, Hitachi Co. Ltd., Tokyo, Japan) and transmission electron microscopy (TEM, FEI Co., Hillsboro, OR, USA) inspections were conducted by using Hitachi SU8010 and Tecnai G2 F20 instruments, respectively. Raman spectra were performed on a Horiba Scientific Raman spectrometer employing the laser line of 532 nm as the excitation source. X-ray photoelectron spectra (XPS, VG Instruments, London, UK) were collected from a VG ESCALAB MARK II apparatus working at 15 kV/300 W using monochromatic Mg Kα radiation source (*hυ* = 1253.6 eV). N_2_ adsorption/desorption isotherms of the corresponding samples were examined at 77 K on a Micromerities ASAP 2020 analyzer, and their specific surface area was measured by the Brunauer–Emmett–Teller (BET) method. X-ray diffraction (XRD, Bruker Co., Karlsruhe, Germany) patterns were recorded on a Bruker D8 Advance diffractometer with a CuKα radiation source (λ = 0.15418 nm) operating at tube voltage of 40 kV and tube current of 40 mA. Thermogravimetric analysis (TGA) was carried out on a Netzsch TG 209F1 equipment by scanning from room temperature to 700 °C in air flow at a heating rate of 5 °C·min^−1^.

### 2.5. Electrochemical Measurements

Electrochemical tests were taken on a CHI 760E electrochemical workstation (Chenhua Co., Shanghai, China) with a three-electrode experimental setup in 2 M KOH aqueous electrolyte solution using nickel foam substrate coated with active material as the working electrode, platinum foil as the counter electrode, and an Hg/HgO electrode as the reference electrode. To fabricate the working electrode, the active material, acetylene black and PVDF were mixed at the weight ratio of 80:10:10. An appropriate amount of NMP was introduced into the mixture, followed by sufficient grinding to form a homogeneous slurry. Then, by coating the resulting slurry onto nickel foam substrate (1 cm × 1 cm), followed by drying at 60 °C overnight in a vacuum oven, the working electrode was generated and the mass of the active materials loaded on each current collector is about 4 mg. Cyclic voltammetry (CV) measurements were done between −1.0 and −0.1 V at varied scanning rates from 5 to 100 V·s^−1^. Galvanostatic charge/discharge (GCD) curves were recorded in the potential range from −1.0 to −0.1 V at different current densities. Electrochemical impedance spectroscopy (EIS, Chenhua Co., Shanghai, China) tests were investigated in the frequency range from 10^−2^ to 10^5^ Hz at open circuit potential with an ac perturbation of 5 mV.

## 3. Results and Discussion

### 3.1. Materials Characterizations

**Figure 2:**
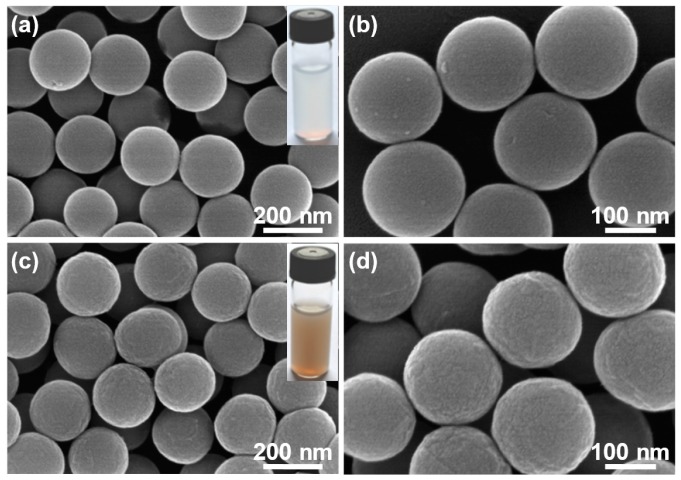
(**a**,**b**) FE-SEM images of pure SiO_2_ microspheres at low and high magnifications, respectively, showing smooth external surface; the inset in (**a**) is a digital photograph of their aqueous suspension, which is pure white in color; (**c**,**d**) FE-SEM images of SiO_2_/GO microspheres at low and high magnifications, respectively, showing relatively rougher outer surface; the inset in (**c**) is a digital photograph of their aqueous suspension, which is yellow-brown in color.

**Figure 3:**
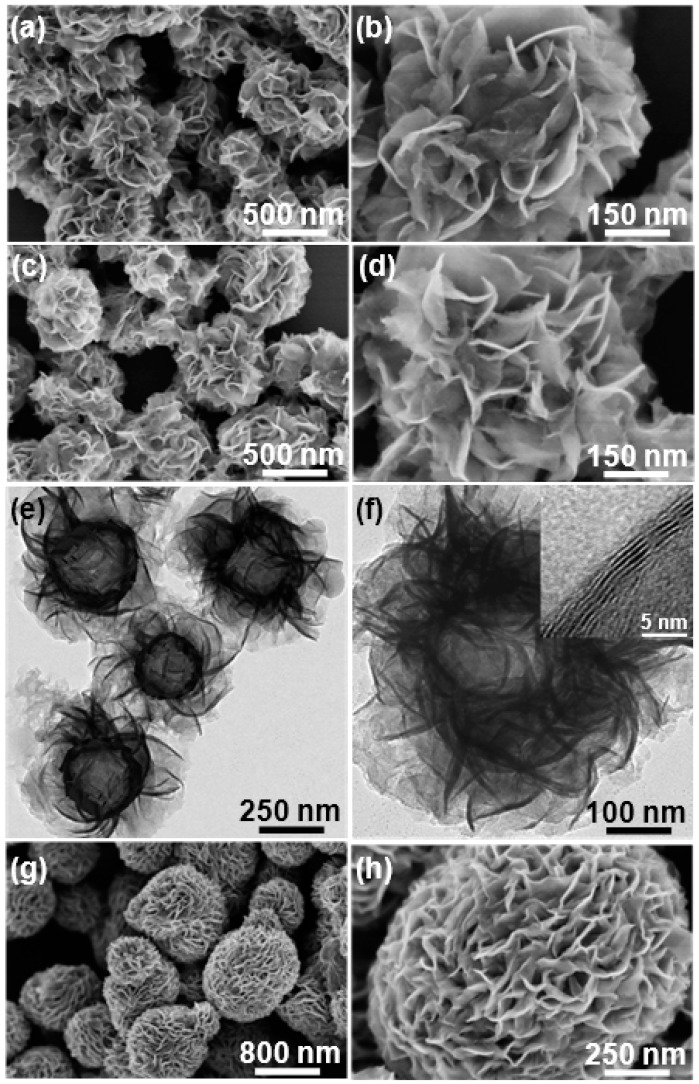

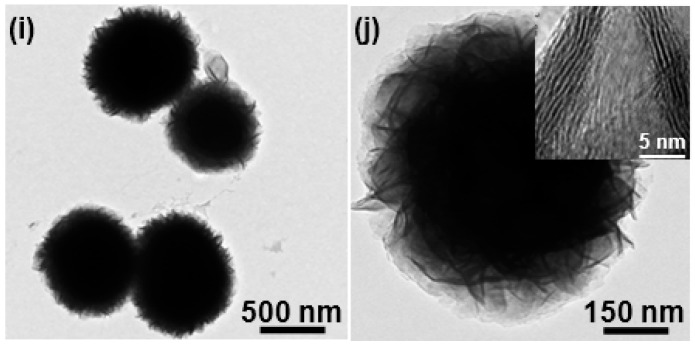
(**a**,**b**) FE-SEM images of SiO_2_/RGO/MoS_2_ microspheres at low and high magnifications, respectively; (**c**,**d**) FE-SEM as well as (**e**,**f**) TEM images of MoS_2_/RGO hollow microspheres at different magnifications; the inset in (**f**) is an HRTEM image of a random MoS_2_ nanosheet anchored on a MoS_2_/RGO hollow microsphere, showing its (002) lattice plane; (**g**,**h**) FE-SEM as well as (**i**,**j**) TEM images of bare MoS_2_ microspheres at different magnifications; the inset in (**j**) is an HRTEM image of an arbitrary MoS_2_ nanosheet located on a bare MoS_2_ microsphere, showing the (002) lattice plane as well.

Figure 2a,b display FE-SEM images of as-prepared SiO_2_ microspheres with excellent monodispersity. Their size is ~200 nm in diameter and the external surface is quite smooth, exhibiting pure white color (inset of Figure 2a). For SiO_2_/GO microspheres, their size is almost the same as that of SiO_2_ microspheres (Figure 2c,d), while the apparent color turns yellow-brown (inset of Figure 2c). Moreover, they seem to be a little rougher as compared with pristine SiO_2_ microspheres and lots of crumples can be identified on the outer surface (Figure 2c,d), which should be ascribed to the encapsulation of tiny GO sheets on SiO_2_ microspheres. These results demonstrate that the sonication-assisted interfacial self-assembly of tiny GO sheets on positively charged SiO_2_ microspheres via electrostatic interaction was successfully achieved. SiO_2_/RGO/MoS_2_ composite microspheres were synthesized by hydrothermal treatment of SiO_2_/GO microspheres in the presence of Na_2_MoO_4_, CS(NH_2_)_2_ and a small amount of HF. Compared with SiO_2_ microspheres, SiO_2_/RGO/MoS_2_ microspheres become bigger with the size of ~500 nm in diameter and their outer surface consists of a large number of nanosheets with the thickness of several nanometers (Figure 3a,b), which should arise from the uniform deposition and growth of MoS_2_ on the substrate microspheres. SiO_2_/RGO/MoS_2_ microspheres were converted into MoS_2_/RGO composite hollow microspheres by selectively etching the SiO_2_ inner core with excessive HF in another hydrothermal reaction and their morphology was carefully examined. As shown in Figure 3c,d, the size of MoS_2_/RGO hollow microspheres seems to be unchanged and the highly curved and wrinkled MoS_2_ nanosheets were well maintained after the template removal process (Figure 3c,d). Whereas an obvious interior cavity with the shell thickness of ~150 nm is available in each MoS_2_/RGO hollow microsphere as disclosed by the TEM observations (Figure 3e,f), indicating the complete removal of SiO_2_ template without the damage of hollow structure. Inset of Figure 3f presents a high-resolution TEM (HRTEM) image of a single random MoS_2_ nanosheet anchored on a MoS_2_/RGO hollow microsphere, where the lattice fringes are clearly observable and the interplanar spacing is deduced to be 0.62 nm, corresponding to the (002) crystal plane of MoS_2_ [17,28,29]. Additionally, contrastive bare MoS_2_ microspheres were also hydrothermally fabricated in the presence of Na_2_MoO_4_, CS(NH_2_)_2_ and a small amount of HF. As exhibited in Figure 3g–j, the sample is almost regularly sphere-shaped in the diameter range from 500 nm to 1 μm and the external surface of bare MoS_2_ microspheres is composed of folded and intertwined MoS_2_ nanosheets as well. However, compared with MoS_2_/RGO hollow microspheres, the bare MoS_2_ microspheres areless porous and hierarchical level with only a solid construction.

**Figure 4:**
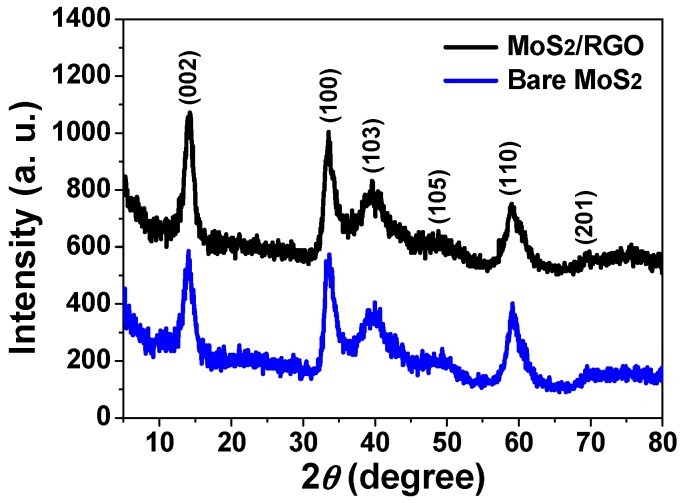
XRD patterns of MoS_2_/RGO hollow microspheres (black curve) and bare MoS_2_ microspheres (blue curve).

To interpret the phase and structural information of the final product, XRD characterization was made. As displayed in Figure 4, six diffraction peaks centered at 2*θ* = 14.3°, 33.6°, 40.1°, 49.1°, 59.0°, and 69.4° are visible in both XRD patterns of MoS_2_/RGO hollow microspheres and bare MoS_2_ microspheres, which are well indexed to (002), (100), (103), (105), (110), and (201) crystal planes of hexagonal phase MoS_2_ (JCPDS no. 37-1492), respectively [6,11,30,31]. In previous reports, RGO gives a typical broad peak at 2*θ* = 25°, nevertheless, it is not found in the XRD pattern of MoS_2_/RGO hollow microspheres, whose absence is probably caused by the weak diffraction intensity and low content of the RGO component incorporated in the final product [20,21,32].

**Figure 5:**
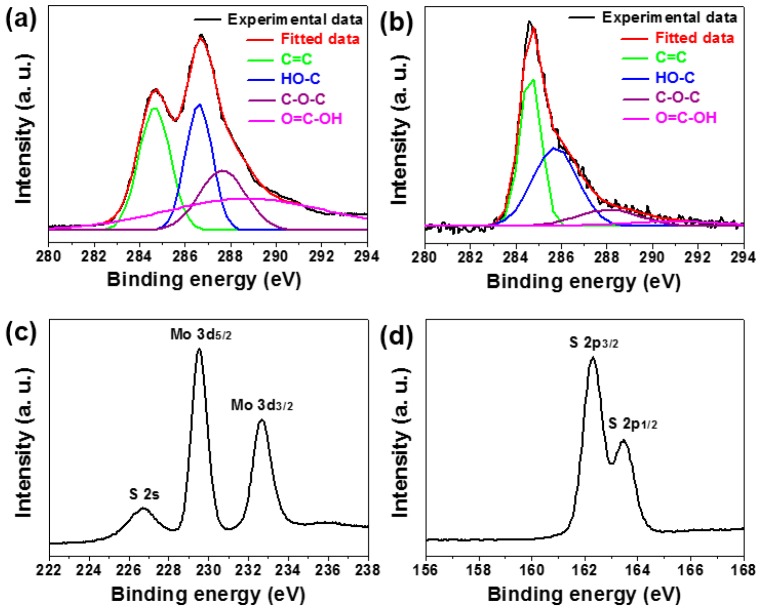
(**a**) High-resolution XPS spectrum of tiny GO sheets, showing the C 1s region; (**b**–**d**) High-resolution XPS spectra of MoS_2_/RGO hollow microspheres, exhibiting C 1s, Mo 3d, and S 2p regions, respectively.

XPS spectroscopy is a powerful characterization tool for surveying the chemical state and composition of hybrid materials. Figure 5a presents the high-resolution XPS C 1s spectrum of tiny GO sheets. Figure 5b–d is a series of high-resolution XPS spectra of MoS_2_/RGO hollow microspheres for C 1s, Mo 3d, and S 2p regions, respectively, and as expected, the detected signals testify the existence of the three elements in the final product. Both of the C 1s spectra are resolvable into four separate Gaussian fitted peaks. The peak centered at 284.6 eV results from the conjugated sp^2^ C=C bonding in graphitic structure, while the other three peaks located at 286.5 eV, 287.8 eV, and 289.2 eV are assigned to multifarious oxygen-containing functional groups such as HO–C, C–O–C, and O=C–OH, respectively [20,33]. Compared with the C 1s spectrum of GO sheets, the relative intensities of peaks for the oxygen-containing groups in the C 1s spectrum of MoS_2_/RGO hollow microspheres diminish dramatically, implying that the GO sheets coated on substrate microspheres underwent an abundant removal of oxygen-containing functional groups and werethus reduced to RGO component during the hydrothermal reactions [20,33]. XPS, Mo 3d, and S 2p spectra reveal representative curves for their doublets with the binding energies (BE) of 229.5 eV for Mo 3d_5/2_, 232.7 eV for Mo 3d_3/2_, 162.3 eV for S 2p_3/2_, and 163.5 eV for S 2p_1/2_, respectively, which agree well with the values for other MoS_2_-based composites reported previously [3,25,34]. The signal of S 2s centered at 226.7 eV is also found in the Mo 3d spectrum, and the BE splitting of 3.2 eV for Mo 3d doublets as well as the BE splitting 1.2 eV for S 2p doublets manifest that the chemical states of elements Mo and S within MoS_2_/RGO hollow microspheres are +4 and −2 valence, respectively [3,25,34]. Namely, they exist in the form of Mo^4+^ and S^2−^, which should come from S–Mo–S bonds [25,34].

**Figure 6:**
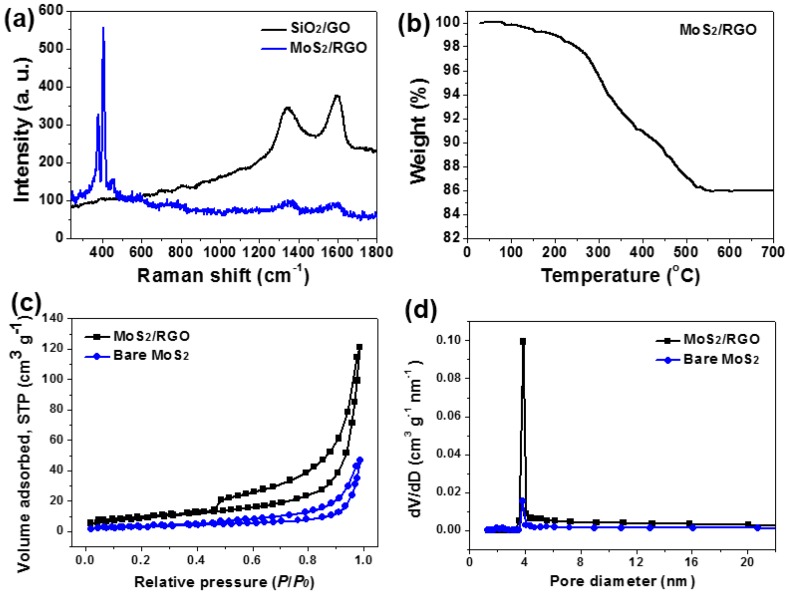
(**a**) Raman spectra of SiO_2_/GOmicrospheres (black curve) and MoS_2_/RGO hollow microspheres (blue curve); (**b**) TGA profile of MoS_2_/RGO hollow microspheres; (**c**) N_2_ adsorption–desorption isotherms; as well as (**d**) pore size distributions of MoS_2_/RGO hollow microspheres (black curve) and bare MoS_2_ microspheres (blue curve).

Figure 6a depicts the Raman spectra of SiO_2_/GO microspheres and MoS_2_/RGO hollow microspheres. Apparently, two characteristic bands located at ~1350 and ~1590 cm^−1^ are available in both curves, which correspond to D and G bands of carbon species, respectively [5,20,21]. For GO- and RGO-based hybrid materials, D band is usually related with the structural defects of symmetrical hexagonal graphitic lattice, whereas G band is derived from first-order scattering of E_2g_ phonons [5,17,21]. Particularly, the Raman peak intensity ratio of D to G band, *I_D_*/*I_G_*, can be utilized to evaluate the defect level within aromatic sp^2^-bonded carbon atom domain [5,17,21]. The value of *I_D_*/*I_G_* for MoS_2_/RGO hollow microspheres is 1.095, which is slightly higher than that for SiO_2_/GO microspheres (0.915), once again verifying the reduction of GO to RGO during the hydrothermal processes [17,21,33]. Apart from the D and G bands, another two dominant peaks centered at ~380 and ~404 cm^−1^ are observed in the Raman spectrum of MoS_2_/RGO hollow microspheres, which should be assigned to the in-plane $E_{2g}^{1}$ and out-of-plane A_1g_ vibrational modes of hexagonal MoS_2_ crystal, respectively [5,6,35]. All these above-described data and results commendably confirm the hybridization of RGO with MoS_2_ to form the final MoS_2_/RGO composite hollow microspheres.

To determine the content of RGO incorporated in MoS_2_/RGO hollow microspheres, TGA analysis from room temperature to 700 °C in flowing air was conducted and the profile was presented in Figure 6b. Supposing that MoS_2_ constituent was completely oxidized by oxygen to produce MoO_3_ and all of the RGO component was burnt out, the ultimate residue should be only MoO_3_, whose weight percentage is 86 wt% [36,37]. Accordingly, the contents of RGO and MoS_2_ within the final product are readily calculated to be approximately 4.5 and 95.5 wt%, respectively.

The porous nature of MoS_2_/RGO hollow microspheres and bare MoS_2_ microspheres were investigated by BET measurements. As shown in Figure 6c, both of the two samples give the N_2_ adsorption–desorption isotherms with a typical hysteresis loop at the relative pressure between 0.45 and 1.0 in each of them. According to the IUPAC nomenclature, such isotherms can be classified into type IV sorption behaviors, suggesting the mesoporous structural characters [6,13,17]. The plots of the corresponding pore size distributions calculated by the Barrett–Joyner–Halenda method are presented in Figure 6d, which once again verify the existence of well-developed mesoporosity in both specimens with rather narrow pore size distribution centered at about 4 nm [6,13]. However, the deduced specific surface area of MoS_2_/RGO hollow microspheres (63.7 m^2^·g^−1^) is much higher than that of bare MoS_2_ microspheres (19.9 m^2^·g^−1^), demonstrating their more hierarchically porous architecture, and the result is quite consistent with the FE-SEM and TEM inspections (Figure 3). The enhancement of specific surface area would enlarge the contact area between the electrode material and electrolyte, offer more reactive sites for electrochemical reaction, and facilitate the transportation of ions and electrons within electrode material, thus ensuring the remarkable supercapacitive behaviors of MoS_2_/RGO hollow microspheres [3,6,13,17].

### 3.2. Electrochemical Tests

**Figure 7:**
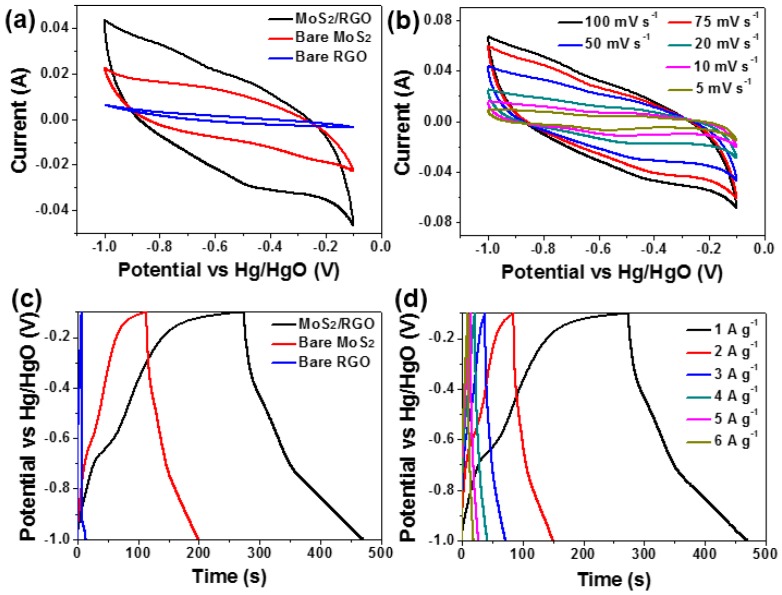

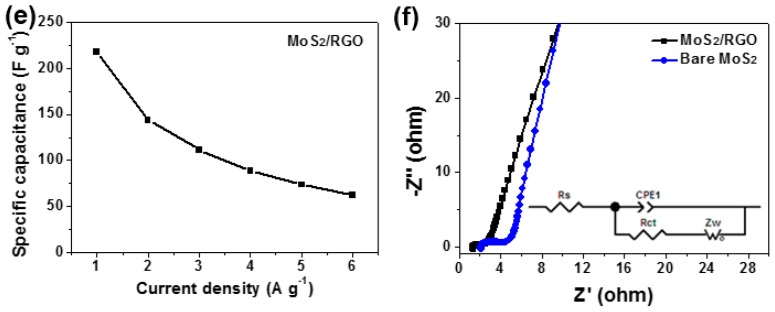
(**a**) CV curves of MoS_2_/RGO hollow microspheres, bare MoS_2_ microspheres and bare RGO material at the sweeping rate of 50 mV·s^−1^ in 2 M KOH; (**b**) CV curves of MoS_2_/RGO hollow microspheres at different scanning rates in 2 M KOH; (**c**) GCD curves of MoS_2_/RGO hollow microspheres, bare MoS_2_ microspheres, and bare RGO material at the current density of 1 A·g^−1^; (**d**) GCD curves of MoS_2_/RGO hollow microspheres at varied current density ranging from 1 to 6 A·g^−1^; (**e**) C_s_ of MoS_2_/RGO hollow microsphere electrode obtained from the GCD curves shown in (**d**) as a function of current density; (**f**) Nyquist plots of MoS_2_/RGO hollow microsphere (black curve) and bare MoS_2_ microsphere electrodes (blue curve) tested in 2 M KOH in the frequency range from 10^−2^ to 10^5^ Hz; the inset is the equivalent circuit used to fit the Nyquist spectra.

To evaluate the electrochemical properties of MoS_2_/RGO hollow microspheres, bare MoS_2_ microspheres, and bare RGO material, CV measurements were performed in 2 M KOH at the same sweeping rate of 50 mV·s^−1^ (Figure 7a). There are no evident redox peaks in all the CV curves, which are similar to previous reports and indicative of the dominant EDLC capacitance feature [3,6,11,13]. The area covered by the CV curve of MoS_2_/RGO hollow microspheres is much larger than that enclosed by bare MoS_2_ microspheres and bare RGO material, implying its enhanced capacitance [5,6]. The CV curves of MoS_2_/RGO hollow microspheres obtained at different scanning rates ranging from 5 to 100 mV·s^−1^ are displayed in Figure 7b. As the sweeping rate goes up, the shape of the CV curves remains good enough, demonstrating that MoS_2_/RGO hollow microsphere electrode possesses excellent charge collection ability and ideal capacitive behavior [3,38]. Figure 7c is the GCD plots of MoS_2_/RGO hollow microspheres, bare MoS_2_ microspheres, and bare RGO material measured from −1.0 to −0.1 V at a constant current density of 1 A·g^−1^. Impressively, the discharge time of MoS_2_/RGO hollow microsphere electrode is remarkably longer than that of bare MoS_2_ microsphere and bare RGO material electrodes, once again manifesting its preferable capacitance, and such GCD performances are in accordance with the results of CV tests. The specific capacitance of single electrode can be obtained based on the following equation:

C_s_ = It/ΔVm

where C_s_ (F·g^−1^) is the specific capacitance, I (A) is the constant current, t (s) is the discharge time, ΔV (V) is the potential window, m (g) is the mass of active electrode material [3,4,6]. Consequently, the C_s_ of MoS_2_/RGO hollow microspheres is calculated to be 218.1 F·g^−1^ at the current density of 1 A·g^−1^, which is not only pretty higher than that of bare MoS_2_ microspheres (94.6 F·g^−1^) and bare RGO material (8.1 F·g^−1^) prepared in this work but also superior to that of other recently reported MoS_2_-based materials such as flower-like MoS_2_ nanostructures, mesoporous MoS_2_ material, hollow MoS_2_ nanospheres, nanostructured MoS_2_ cluster, MoS_2_/C, MoS_2_/SnS_2_, MoS_2_/SnO_2_ composites and so on [3,6,11,12,13,14,15,16]. Figure 7d depicts the GCD curves of MoS_2_/RGO hollow microsphere electrode at different current densities and the variation of its C_s_ with current density is plotted in Figure 7e. As can be seen, the C_s_ of MoS_2_/RGO hollow microspheres gradually decrease as the current density increases from 1 to 6 A·g^−1^. That is because both the inner and outer active sites and pores of electrode material would be sufficiently accessed by electrolyte ions at low current densities, leading to high C_s_ values, whereas only the external surface of electrode material contributes to charge/discharge processes at high current densities, hence bringing about the reduction of the C_s_ [3,39]. It is assumed that two reasons are responsible for the prominent capacitive property of MoS_2_/RGO hollow microspheres. On one hand, the hybridization of MoS_2_ with RGO minimizes the overall electrode resistance, thus improving its electric and ionic conductivity. As shown in the Nyquist plots (Figure 7f) obtained from the EIS measurements of MoS_2_/RGO hollow microspheres and bare MoS_2_ microspheres, each curve consists of an arc in the high-frequency region and a spike in the low-frequency region. The experimental data can be simulated by an equivalent circuit model (inset of Figure 7f), where R_s_ represents the internal resistance, CPE1 is a constant phase element for the electric double-layer capacitance, R_ct_ is the charge transfer resistance, Z_w_ is the Warburg impedance [6,29]. R_s_ is associated with the ionic resistance of electrolyte and electronic resistance of electrode, which is able to be deduced from the intersection of Nyquist plot on the real axis (Z’), while R_ct_ is ascribed to the electrochemical process taking place at the interface of electrolyte/electrode, which can be directly obtained from the diameter of semicircular arc [6,29]. The R_s_ value is 1.35 Ω for MoS_2_/RGO hollow microspheres, which is lower than that of bare MoS_2_ microspheres (2.04 Ω); the R_ct_ value of MoS_2_/RGO hollow microspheres is 0.61Ω, which is also smaller than that of bare MoS_2_ microspheres (1.85Ω). These results unravel that the RGO component indeed serves to reducethe resistance of MoS_2_/RGO hollow microspheres, thereby enhancing the conductivity and capacitance. On the other hand, MoS_2_/RGO hollow microspheres feature unique porous hollow architecture with high-level hierarchy and large surface area, which expands the electrode/electrolyte interface area and is beneficial to the fast transport of electrolyte ions throughout the electrode matrix during the charge/discharge processes, further elevating the electrochemical capacitive performance.

**Figure 8:**
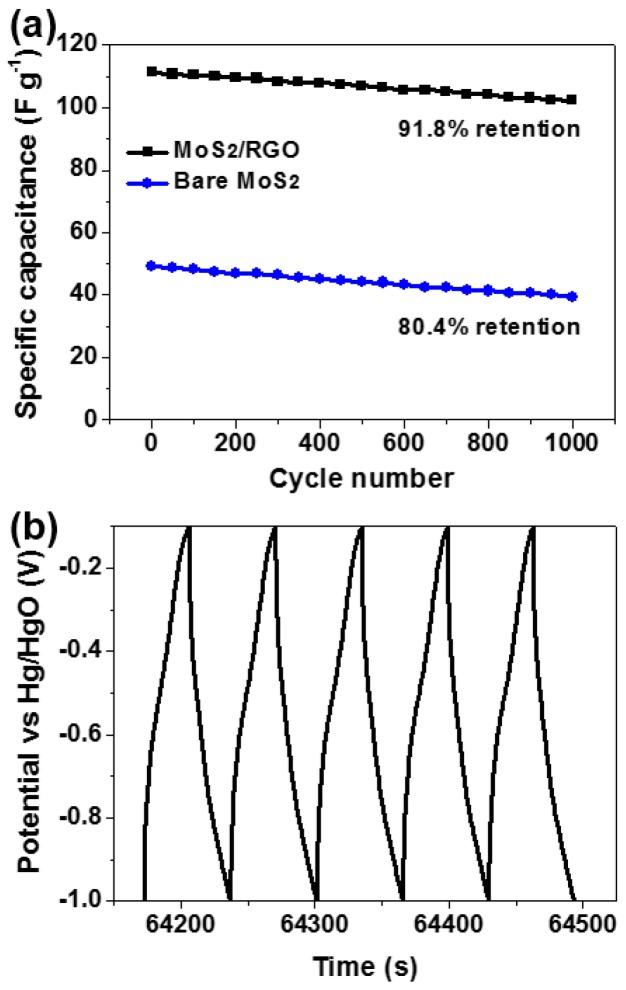
(**a**) Cyclic performances of MoS_2_/RGO hollow microsphere (black curve) and bare MoS_2_ microsphere (blue curve) electrodes at the current density of 3 A·g^−1^ in 2 M KOH; (**b**) The last five cycles of charge/discharge curve for MoS_2_/RGO hollow microsphere electrode.

The cycle life is one of the most crucial parameters for practical application of supercapacitors. The cyclic performances of MoS_2_/RGO hollow microsphere and bare MoS_2_ microsphere electrodes are examined by GCD tests for 1000 cycles at a constant current density of 3 A·g^−1^ (Figure 8a). As the cycling proceeds, the C_s_ of the former decays quite slowly and is always larger than that of the latter. Moreover, the shape of the last five charge/discharge curves of the MoS_2_/RGO hollow microsphere electrode is nearly unaltered (Figure 8b), and its C_s_ retention still reaches up to 91.8% after 1000 cycles, which is better than that of bare MoS_2_ electrode (only 80.4% retention), demonstrating that the hybridization of MoS_2_ with RGO enhances the stability and durability of the electrode material.

## 4. Conclusions

In conclusion, SiO_2_/GO composite microspheres were fabricated by sonication-assisted interfacial self-assembly of tiny GO sheets on positively charged SiO_2_ microspheres, which were then employed as the template to hydrothermally develop MoS_2_/RGO composite hollow microspheres. It was found that MoS_2_/RGO hollow microspheres had distinct porous hollow architecture with more hierarchical level and larger specific surface area as compared with bare MoS_2_ microspheres, which were also hydrothermally prepared under identical conditions except for not using the template. Thanks to the unique structural and morphological features as well as excellent conductivity, MoS_2_/RGO hollow microsphere electrode released a maximum specific capacitance of 218.1 F·g^−1^ at the current density of 1 A·g^−1^ and maintained 91.8% of the initial capacitance after 1000 charge/discharge cycles at the current density of 3 A·g^−1^, exhibiting saliently supercapacitive advantages over the currently synthesized bare MoS_2_ microspheres and most other reported MoS_2_-based materials. Moreover, by virtue of the convenience and versatility of the methodology presented in this work, it would be viable to achieve the sonication-assisted interfacial self-assembly of tiny GO sheets on other solid substrates with different composition and shape. Accordingly, it is believed that through appropriate modification of the resulting GO coated hybrid materials with functional organic or inorganic species, a large variety of advanced GO- and RGO-containing composites with diverse structures and enhanced properties will be explored, which would find applications in many important fields such as energy storage, biological separation, water treatment, photocatalysis, and so on.
